# Inaugural Thesis on the Action of Remedies

**Published:** 1881-03

**Authors:** Robert O. Cotter


### INAUGURAL THESIS OF ROBERT O. COTTER, ON THE ACTION OF REMEDIES.

For which the prize of a medical case was awarded at the Annual Commencement of the Atlanta Medical College, March 2, 1881.

Under the general name of remedies, are included the various means applicable to the relief or cure of disease. These various agents are known as medicinal, psychical, surgical and mechanical remedies. Lack of space forbids my considering each of these classes in this brief essay, and consequently, I shall only endeavor to present a brief outline of the action of some of our important *medicinal* remedies.

There have been, more or less, in all ages two systems of medicines, viz: The *empirical*, which prevails among ignorant men, or in rude states of society; and the *rational*, which requires a higher degree of enlightenment. The former is founded upon a very simple system of reasoning. By accident or experience it is found that a certain medicine is of use in the treatment of a certain disorder, and on a number of such separate data, the empirical system is founded, and which naturally requires a small degree of knowledge for its elaboration.

This observation of facts is of course highly necessary as a beginning, but much more is required to make our plan of treatment a rational one. We must not be content with taking these facts separately, but must proceed to compare together a large number of them, and then draw inference from this comparison, and thus when on the one hand from an accurate knowledge of the symptoms of disease, we are enabled to meet each by its appropriate remedy, and on the other hand, from accurate acquaintance with the general action of a medicine, we are enabled to wield it with more skill and effort, and to apply it even in cases where it has not yet been proven beneficial.

When our acquaintance with these two shall have become complete, shall we not then be able to do all that man can by any possibility effect in the alleviation of human suffering? Diagnosis and nosology are making rapid strides, but it must be confessed that therapeutics in spite of earnest laborers and able investigators, is making but indifferent progress.

Various and amusing to think of at the present time are the theories in past ages as to how medicines acted; on no question perhaps have scientific men differed more. One class of the ancients contended that medicines acted simply mechanically, that is, many diseases of the solid parts were attributed to a weakness or laxity of the animal fibres, and that medicines acted mechanically by increasing their tenacity. Some supposed that some medicines, as for instance, mercury and arsenic, the former from its particles being round and heavy, were able, when shaken about in the vessels, to break up and annihilate acrid humors which were the cause of disease, and that arsenic acted as an irritant by the sharp and jagged nature of its particles.

A court physician, in Europe, as late as 1751, feared that the administration of mercury was dangerous in cases where there was carious bone, lest its heavy particles might break the weak lamellæ.

But it is much easier to find fault than it is to preach, and we should recollect that the best way to arrive at the truth is by being extremely careful in the means which we employ in its discovery.

In regard to the action of medicines, I shall attempt to present a general idea of those I shall speak of as regards—

1. Their mode of operation,
2. As to what organ or tissue their action is directed.
3. The result of their action.

As regards classification, lack of space precludes anything more than a very general classification here, and I think it would not be incorrect to say, that medicines might be grouped in four great classes, the action of which is well marked and distinct; as a matter of course, many special medicines are known to partake of the nature of more than one class, and of which probably more will be said towards the close of this essay.

These four classes I shall designate as:

1. Hæmatics.
2. Neurotics.
3. Astringents.
4. Eliminatives.

Of *hæmatics* or blood medicines—as a large number of diseases depend upon a fault in that fluid—it is said we may by their means be enabled to remedy that evil. These are consequently among the most important of remedies.

Hunger, properly considered, is itself a disease. It is a demand from the blood for the renovation of its failing constituents; a demand for a fresh supply from the body, which is always changing and always requiring nutriment. A proper article of food may be taken as a type of what may be styled *restorative hæmatics*.

Restorative hæmatics act while in the blood; they are natural to the blood, that is they coincide with certain substances that exist in the body, and are consequently not excreted.

The action of hæmatics is of a slower nature than that of neurotics. When from any given cause, the system is badly nourished, and the blood lacking the important constituents, nourishing food containing the proper amonnt of components required, say albumen, fibrine, oiley matters, etc., becomes a valuable medicine.

Prominent among this division may be mentioned—Butter, eggs, flour of meat, cod liver oil, and others, such as bread and vegetables, containing the various salts of potash, lime, etc., the phosphates, sulphur, chloride of sodium, etc.

*Iron* is a prominent restorative hæmatic. It is a well known constituent of the blood, being found in that fluid or tissue which partly constitutes the coloring matter of the red corpuscles, which is called hæmatosin; when this is deficient it is evinced by the paleness of the tissues in anæmia and great general debility of the whole system. Iron is adapted to the cure of anæmia, and is apt to be beneficial in other disorders in which anæmia is a prominent symptom, such as amenorrhœa, scrofula, cancer, chronic ague, hysteria, etc.

*Catalytic hæmatics*.—Besides the foregoing class of hæmatics, I shall speak of a class of hæmatics which, though they enter the blood, are not natural constituents of that fluid, and would, if they remained in it, prove injurious to it, and hence must be and are excreted by the glands. They are of use when disease depends upon the presence, and working in the blood of some morbid or poison material, which they tend to counteract and destroy. These then are called catalytic hæmatics, from a Greek word, which signifies a break up or destroy, and they having performed their function, pass out of the system.

I shall only briefly consider the action of one medicine of this division, viz: *mercury*.

It is a valuable antiphleogistic in cases of inflammation and inflammatory fevers. In these disorders there appears to be an excess of those natural elements of the blood upon which nutrition depends, say an excess of fibrine in the blood, which is, of course, a morbid condition, and unless checked in time tends to cause inflammatory effusions and painful adhesions, as in pleuritis metritis, peritonitis, etc., or if the fever is kept up it may exert such a powerful influence over the nervous system as to produce death. Mercury has the direct power of meeting and decomposing, and thus depriving the blood of about one-third of its fibrine, and thus proves to be a very valuable antiphleogistic.

But as a catalytic, it is in the treatment of another disorder that mercury stands at the head of the list as a specific, and this is in syphilis. This disease, it is well known, is the result of a specific poison taken into the blood either by direct innoculation or transmitted by inheritance, which if left alone, breeds in the system, and after effecting all the mischief it is capable of may be propagated to others in the same manner in which it was contracted.

In the primary stage of this disorder we know that mercury is the best remedy that we can make use of, but as to the precise manner of action of mercury here it is not positively known. This catalytic action is shrouded in great mystery. We may proceed along step by step in this interesting investigation, and when we think we have arrived at the end of our work we find that we are utterly unable to take the last step. It would, perhaps, be best to confess our inability and say we only know the therapeutic *result* of some of the medicines of this division, and yet it is said there is a tendency in the human heart to seek to explain everything, and should I be thought guilty of explaining more in the course of this essay than I know, I shall plead this weakness as an excuse. It would seem likely, however, that syphilis, with the eruptions, ulcerations and neuros s which result from its workings, is obviously an agent which tends, sooner or later, to impoverish the blood, and it is reasonable to suppose that mercury in its destructive action may first seize upon those parts of the blood which are most diseased or most liable to decomposition, and that it may thus grapple immediately with the fermenting virus of syphilis as well as those materials of the blood upon which it has commenced to prey. (Like a retreating army destroying all the means of subsistence before an invading host, hoping thereby to check their further advance). Mercury is of prime importance in the earlier stages of syphilis, but may be administered in the latter stages in cases where its use has been omitted in the primary. Here iodine, or its salts, should be given, for it seems that after the poison has been met and modified it is but capable of being controlled by iod.-potash.

Regretting that lack of space precludes a possibility of the consideration of more valuable agents of this division, such as iodine as an anti-scrofulous catalytic and others.

The next or second class of medicines, *neurotics*, will be considered. Neurotics are medicines which influence the nervous system by exciting, depressing or otherwise altering its tone. They are chiefly useful in the temporary emergencies of acute disorders. If the action of catalytics was difficult to understand, how much more incomprehensible is the action of neurotics. I should be satisfied here if I could explain *what* their action is, and leave it to abler investigators to say how their agency is effected. The action of a neurotic, unlike that of a hæmatic, is rapid; does not cause any change in the blood, but passes out.

Most of the powerful poisons which act after passage into the blood are of this class. Death may be produced by one of them, but we find upon an examination no apparent cause for this. No perforation of the coats of the stomach or intestines. No laceration of nerve fibre. How then could death have been produced?

This class may be considered under three divisions:—

1st. Nervous stimulants.

2d. Narcotics.

3d. Nervous sedatives.

Of *stimulants*—they vary much and exalt nervous force, either of the whole nervous system or a part of it. Prominent as a stimulant I shall speak of ammonia. Suppose a case of syncope, which most likely depends upon a shock received by the brain. Several things go to show that the efficacy of ammonia in this condition is due to its action exerted upon the cerebral centre more especially than upon the heart and vascular system, in which it is found to be more prompt and effective when administered through the medium of the lungs than by the stomach.

Take also a case of poisoning by snake bite. In this condition the nervous centres are depressed, and *ammonia*, by exalting nervous force, proves the best of stimulants.

*Narcotics*, the second division of neurotics, have a peculiar influence over the functions of the brain, which is different from any possessed by other nerve medicines. They control the *intellectual* part of the brain more especially than its organic functions, that is, the powers of mind more than those of life. Some of this division produce *intoxication*, some *sleep*, and some *delirium*. I shall consider narcotics principally as regards their secondary action, as the majority of them produce primarily exaltation of nervous force. They, as remarked previously, principally influence the intellectual functions; the functions by which the mind is connected with the body, and the five senses, by which the mind, through the body, is connected with external impressions. Given in over doses they produce coma, gradual suspension of respiration and death.

As an *intoxicating* narcotic, I shall speak of *alcohol* as a type of its division. Alcohol is applicable as a medicine in asthenic fevers, typhoid forms of pneumonia, in prostration and collapse, the result of injuries, etc.

The stimulant effect restores the action of the heart and enables the system to bear up against the disease. The power that alcohol possesses in retarding tissue metamorphosis, is well known and appreciated.

Aside from the terrible moral degradation and social ruin, which its continued use may bring about, and of which it is not my province to speak here, its excessive use may, besides producing chronic injury of the brain and mind usually resulting in delirium tremens, in over doses produce coma and death.

As a sleep-producing narcotic opium heads the list. Like alcohol, opium primarily produces exaltation of nervous force; it then produces drowsiness and sleep. The condition of sleep is a peculiar one, in it the senses of sight, hearing, smell and taste, and, to a considerable extent, touch are subdued; it may be interrupted by a slight physical shock or a forcible impression made upon one of the senses which are held in subjection. But this condition is different from intoxication and delirium in that except in rare instances the latter two cannot be put an end to in this way.

Pain prevents sleep, and a dose of opium may prove an anodyne and relieve pain, and yet it may prove an anodyne independent of its sleep-producing power; it may produce relaxation of the muscular system, and this prove an antispasmodic, and under its action the whole material nervous system is quieted and controlled. The chief indications for its use are pain, wakefulness and nervous excitability.

A large dose of opium causes paralysis of the radiating fibres of the iris, and this brings about contraction of the pupil; a larger dose may cause coma, suspension of respiration and death.

The third order of the division of narcotics produce *delirium*, and I shall mention one prominent medicine of this kind, viz., *belladonna*. Its brief primary action is to produce a quickness of pulse and a slight heat of skin; the secondary effects are to produce an anodyne effect and a sedative influence upon the heart and circulation. It is useful in painful disorders as fevers and inflamations; also as an anticonvulsion, for instance in pertussis and other spasmodic coughs. Its alkaloid, atropia, is of great use in some eye affections and in surgical operations upon the eye, applied externally. It causes dilatation of the pupil (just the opposite of opium), by paralyzing the sphincter fibres of the iris, and in connection I may remark, that in cases of poisoning by either of these medicines, one is said to be an antidote for the other. But it is more than probable, however, that much of the truth of this remains yet to be proven.

Opium also checks intestinal secretions, by decreasing peristaltic motion of the bowels, while belladona increases them by increasing peristaltic motion, besides being antagonistic to each other.

Belladonna in large doses, as before remarked, produces dilatation of the pupils, great dryness of the mouth and fauces, often erythematous eruption, and in still larger doses, delirium, exciting and leading astray the mind, presenting unusual objects and hallucinations to the senses; poisonous doses lead to convulsions and coma, ending in death.

Some of the physiological effects are quite vivid to my own mind from personal experience. During our session of 1879 and 80, I took by mistake for another medicine, probably five grains of the ex ract at bed time, and soon after went to sleep. I awoke two hours afterwards as if from a troubled dream, and feeling very much bewildered, with a most distressing dryness of the mouth and fauces, and unsteadiness of limbs, could scarcely manage to light my lamp, and nearly blind from dilatation of pupils, after procuring medical assistance and getting relieved of the immediate danger of the poison. Next day being anxious to read up its physiological action, I found it utterly impossible for the period of twenty-four hours after taking it, to see to read one word or write my name; flashes of beautiful colored light was all I could see upon making any attempt to examine small objects; vesical paralysis was also for a few hours a prominent symptom in my case.

*Sedatives*, the third division of neurotics, tend to depress nervous force. They may act upon the whole nervous system or a part of it only. They are of use when from any cause some part of the nervous system is over-excited.

Unlike stimulants, they do not produce any primary exaltation of nervous force, and also in fatal doses, instead of producing death by coma, as in the case of stimulants, they produce death by syncope.

Neither do they exert any special influence upon the intellectual part of the brain.

As a prominent medicine of this division, I shall speak of hydrocyanic acid. It is a most powerful sedative to the nerves generally; it acts as an anodyne by producing insensibility and controlling muscular action; it is of use as an anti-spasmodic. It also appears to influence reflex nervous action; it, therefore, is of use in the treatment of whooping cough and asthma, which have a reflex origin from the irritation of the mucous surface. It is also of use in cardialgia, by diffusing itself through the mucous membranes of the stomach, and coming in contact with the irritated nerves at all points, tend to allay this pain. It also acts upon the superficial nerves, upon being applied to the skin. It is a most dangerous medicine; inhalation of its vapor will produce death; a single drop of it undiluted is sufficient to destroy life. It must only be administered in a largely diluted form, and even in this way it may produce slowness of pulse. Loss of muscular power, spasms, dilated pupils, leading to loss of consciousness, collapse, syncope and death.

*Astringents.*—The third class of medicines are those which act upon muscular fibre, causing it to contract. They chiefly act upon the involuntary or unstriped muscular fibre, which is not directly controlled by the brain and nerve centres, and for this reason are more under control of external or irritating agents. Involuntary muscular fibre exists in the coats of small blood vessels and ducts of glands, and astringents are able by contracting muscular fibre, to diminish the calibre of these vessels, and thus in one case when a small blood vessel is ruptured arrest hemorrhage, and in another case prevent the outpouring of a secretion. Their action is comparatively transient. They seem to possess both a chemical and a dynamical force. The chemical power is evinced by their power of coagulating albumen and fibrine, which power is manifested outside the body as well as within, and the dynamical power is that which they exert in causing contraction of muscular fibre. The chemical action is clearly evinced by the fact that they are best when applied directly to the part to be acted upon, and least when diluted with the whole mass of the blood.

As to the dynamical force, by means of the chemical combination with the fibrine of the muscular fissue, it appears that this constriction thus set up chemically is continued and propagated by the vital force of the muscle. As this class all act pretty much alike, I shall only endeavor to illustrate their use by one prominent vegetable astringent, *tannin*. This medicine and its modification, *gallic acid*, into which it is converted when taken into the system, proves of great benefit in the treatment of hemmorrhage from the lungs, stomach, and nose, prolapsus, balanitis, leucorrhœa, and night sweats depending upon phthisis.

It is a very innocent medicine, and a large dose may only cause a feeling of dryness of the mouth and fauces and may excite some pain in the bowels and stomach and slight constipation. As the action of this class is so simple I shall not dwell longer upon them but proceed to a consideration of the next grand group, *eliminations*, which have the power of increasing the secretions which are formed from the blood by various glands at different parts of the body.

By their aid we may be enabled to eliminate from the blood a morbid material through the glands or to do great good by restoring a secretion unnaturally suppressed. As a rule eliminations are substances which are unnatural to the blood and must in most instances pass out of it and in doing so it seems that they tend to pass out by some glands rather than others, and in the secretion of these may be detected chemically, and on these glands they appear to have an especial or elective influence. Thus we find that cathartics are excreted by the bowels, diuretics by the kidneys, diaphoretics by the skin and so on.

The result of this passage through a gland is to increase its secretion and they are of use when any condition of the system requires that the function of a gland should be restored or promoted. There is one general law of secretion which we should consider, and that is the special function of each gland or set of glands to secrete from the blood particular materials and to pass them out of the body. In regard to passing them out, however, there are some exceptions, for instance the bile and some other substances are known to be reabsorbed after being secreted.

The value of evacuants, for instance, has long been appreciated and their efficacy been known as in cases of fever and other disorders; they caused the elimination through the glands of certain matters that were formed in the blood, but ought not to remain in it. Eliminations are probably the most useful of our medicines. They may be useful in restoring the function of a gland when impaired. They may eliminate a poison or morbid material and thus cure disease.

We may by their use replace the function of a diseased gland by eliminating its product from the body by another gland.

They may also be of great use when pushed to further extent, by actually causing the excretion of some of the natural constituents of the blood; as, for instance, by increasing the secretion of the intestinal glands, may thus draw away the serum of the blood, and, as I shall show further on, may actually deprive the blood of a portion of its fibrine, and thus prove valuable antiphlogistics and depletives.

Not having space to notice many medicines which come properly under this division, such as expectorants, sialogogues and others, I shall mention cathartics as being probably the most important of eliminatives. It was for a long time supposed that the fæces consisted only of what remained of the unabsorbed portion of the food, and that purgative medicines acted simply by exciting the peristaltic motion of the bowels, and thus caused the ejection of those unabsorbed matters. But it is now known that fæcal matter is composed in great part of the excrementitious substances which are separated from the blood by the glands of the intestinal mucous membrane, and it is more reasonable to suppose that *true* cathartics are often being absorbed into the circulation, excreted by these intestinal glands which they stimulate, and thus produce catharsis. Here it is proper to say, *some* substances, such as bran, charcoal, etc., which are not absorbed, act as cathartics simply by their mechanical influence. It is generally supposed that the bile will produce cathartics. Among different views as to its manner of action, a late one, I believe, is that being acted upon by a dose of mercury, which meets it in the duodenum, it is rendered insoluble, and not being reabsorbed it passes along the intestinal tube, irritates the mucous surfaces, probably both stimulates the intestinal glands and causes peristaltic motion of the bowels and catharsis.

Yet it is of true or specific cathartics (so to speak) that I shall deal with here.

This class of medicine is named "legion," and greatly varied in their action. Some are simply laxative or mild purgatives, such as rhubarb, aloes, castor oil, etc., and when it is simply desired to evacuate the contents of the bowels in cases of constipation these will generally be found sufficient to answer the purpose.

By way of digression, as I shall not consider the so-called class of emmenagogues, one of the foregoing, viz.: aloes, owes its influence upon the uterus to the propagation of *irritation*, by means of contiguity of structure. Aloes, by acting upon the lower part of the intestinal canal, causes peristaltic motion, which action is extended to the contiguous uterus, which from the irritation and congestion following this action, may cause in one case a reappearance of the catamenial secretion when suppressed, or in the case of a gravid uterus may tend to bring about premature expulsion of its contents, and hence in the latter condition we should be careful in the administration of this purgative.

*Mercury and sulphate of magnesia* are two of our most useful cathartics, sometimes even in the very reverse condition, apparently, in which the use of a cathartic is indicated, as in dysentery a mercurial cathartic, and also Epsom salts prove the best of remedies. In this disorder the function of the excreting glands of the intestines is deranged and pouring out an excessive and unnatural secretion and causing griping pain in the bowels. Mercury, by changing this unhealthy secretion and restoring tone to the deranged glands, relieves this painful disorder.

*Sulphate of Magnesia* as a cathartic may prove to be a valuable antiphlogistic. It is useful in acute fever and inflammation. By a chemical action it has the power of actually controlling and preventing the development of plastic fibrine in the blood, and in speaking of its valuable depletive effects I shall always remember how Prof. John G. Westmoreland sought to impress the class with the fact that "we need not be afraid of the danger of purging a puerperal woman to death, but that she would get stronger and better with each purge."

I shall next say something of *diuretic* eliminations:

These tend to increase the secretion of the urine. In the first place it is necessary that the blood should be kept down to its natural standard as regards water, and it is principally by means of the kidneys that this is effected. Second, There are continually forming in the blood some soluble matters, as urea and uric acid, which, if retained in the blood, act as poisons, and must be eliminated by the kidneys.

There are many diuretics, taking the term in its general acceptation. Simple water is a diuretic in that, by increasing the quantity of fluid in the blood it, of course, increases the flow of the urine. Other substances, such as oil of turpentine and copaiba, which stimulate the mucous membrane generally, tend to stimulate the action of the kidneys simply by means of the intimate connection of the kidneys with the membrane. However, a few, such as cantharides, juniper and savin are free senal stimulants or diuretics; for instance, *cantharides* is taken into the system, its acid stimulating principle cantharodine is rendered soluble by an alkali in the intestine is absorbed, and in the renal apparatus meets with an acid which again decomposes it, and liberates the irritant cantharodine which acts as a powerful renal stimulant. Given in too large doses these diuretics may defeat the very object for which they were administered, for by stimulating the mucous membrane of the urinary apparatus and the kidneys to too great an extent, may produce strangury, congestion of the kidneys, and even suppression of the urine for a time.

In case of congestion of the liver, kidneys or whole venous system, in this pressure of the circulation absorption of water can not go on, and the urine is necessarily diminished, and it would thus seem that diuretics might be most useful remedies, and yet, after, from the reasons just given, while their use may be plainly indicated, we can not obtain the relief from them which it seems they could afford.

I beg pardon for extending this essay to what I suppose will be unpardonable length, but I suppose I cannot make it much worse by yielding to the temptation of saying something of the medicines mentioned in the first part of the paper, which partake of the nature of more than one class.

From all the authorities which I have examined, I think quinine might properly be classed under this head.

It has for a long time been recognized that there existed a bitter principle of the blood; this principle is secreted by the liver, and belongs to that part of the bile which is reabsorbed into the blood from the intestinal canal. Experiments made upon animal blood have demonstrated that a substance, some of whose components are identical with quinine, is to be found in the blood of animals, and it has been ably argued that this may be found in the blood of man. I simply say this has been done, and occupy neutral ground as to my own opinion, but while I am aware of the fact that many of our most intelligent practitioners are disposed to scoff at comparative anatomy, the history of medicine shows what it has done to forward the progress of the science. Quinine is considered a specific for ague or malarial fever; to sum up rapidly the cause, symptoms and pathology of this disease, a peculiar poison or miasm generated by the ground in some places.

The result of this poison taken into the system is a a sort of fermentation, producing regularly recurring paroxysms of a peculiar kind—a disturbance in the great heating process of the body, in which the blood is concerned. Each fit comes on with a shivering, then a hot stage, and finally sweating. The attack then goes off, apparently as if the poison which caused it had been eliminated by the perspiration, but it is not *all gone yet*, for after working in the blood for a certain length of time it again breaks out, and the same train of symptoms again follow. Thus this disorder certainly appears to be in the blood; its results are evinced there by continued ague; deteriorates the blood, producing general anaemia, and also enlargement of the spleen. It is reasonable to suppose that in ague there is a lack of some natural material in the blood, which, if present, would have the effect of checking the operation of the morbid agent. Suppose a number of *healthy* persons are exposed to the virus of small-pox or the fermenting germs of measles, under favorable circumstances, they are all apt to catch these diseases at once, but such would not likely be the result in case of several persons exposed to the poison of malaria. Some escape, and it is generally found that those are most likely to take it who have been debilitated by any cause, and have not in their blood the material which serves to prevent the working of this poison, and should there be any truth in the theory that quinine is identical with some of the components of the blood, does it not then supply this missing principle, and as a haemetic and a curative agent in ague. Besides, really partaking of the nature of nearly all the classes of medicines. It is especially a neurotic. This is shown by the various nervous symptoms which are produced by it. Excessive doses of it produce cinchonism, which is characterized by loud ringing noise in the ears, splitting headache, vertigo, partial deafness or blindness, or even sometimes approaching delirium. It is also an excito and motor tonic, and its stimulating effect upon the uterus renders large and continued doses of it, during pregnancy, particularly hazardous.

